# Toripalimab plus chemotherapy in American patients with recurrent or metastatic nasopharyngeal carcinoma: A cost‐effectiveness analysis

**DOI:** 10.1002/cam4.7243

**Published:** 2024-05-16

**Authors:** Dai Lian, Yuling Gan, Dunming Xiao, Dennis Xuan, Yingyao Chen, Yi Yang

**Affiliations:** ^1^ School of Public Health Fudan University Shanghai China; ^2^ National Health Commission Key Laboratory of Health Technology Assessment Fudan University Shanghai China; ^3^ School of Public Health and Tropical Medicine Tulane University New Orleans Louisiana USA

**Keywords:** cost effectiveness, immunotherapy, nasopharyngeal carcinoma, toripalimab

## Abstract

**Background:**

Toripalimab, combined with gemcitabine and cisplatin, has been approved as the first‐line treatment for recurrent or metastatic nasopharyngeal carcinoma (RM‐NPC), representing a significant milestone as the first FDA‐approved innovative therapy for this condition. Despite this achievement, there's a lack of data on the cost‐effectiveness of toripalimab for RM‐NPC patients in the American context.

**Methods:**

To assess the cost‐effectiveness of toripalimab plus chemotherapy versus chemotherapy alone, a 3‐state partitioned survival model was constructed. The study involved participants with characteristics matching those in the JUPITER‐02 trial. Cost and utility inputs were collected from literature. Main outcomes measured were quality‐adjusted life year (QALY), and incremental cost‐effectiveness ratio (ICER). Univariate and probabilistic sensitivity analyses, subgroup analyses, and scenario analyses were conducted to verify the robustness of results.

**Results:**

The study found that the toripalimab regimen resulted in 4.390 QALYs at a cost of $361,813, while the chemotherapy‐only regimen yielded 1.685 QALYs at a cost of $161,632. This translates to an ICER of $74,004/QALY, below the willingness‐to‐pay threshold of $150,000/QALY. Sensitivity analyses indicated that utility values, discount rate, and the price of toripalimab significantly impact INMB. With an 87.10% probability of being cost‐effective at a $150,000/QALY threshold, the probabilistic sensitivity analysis supports toripalimab plus chemotherapy as a viable option. Scenario analysis showed that toripalimab remains cost‐effective unless its price increases by 125%. Additionally, a simulated 15‐year study period increases the ICER to $88,026/QALY. Subgroup analysis revealed ICERs of $76,538/QALY for PD‐L1 positive and $70,158/QALY for PD‐L1 negative groups.

**Conclusions:**

Toripalimab in combination with chemotherapy is likely to be a cost‐effective alternative to standard chemotherapy for American patients with RM‐NPC. This evidence can guide clinical and reimbursement decision‐making in treating RM‐NPC patients.

## 1 INTRODUCTION

According to the estimations by the International Agency for Research on Cancer, there were 1898 newly diagnosed cases of nasopharyngeal cancer in the United States in 2020, with 915 reported deaths due to this malignancy.^1^ In addition, most patient diagnoses of nasopharyngeal carcinoma in the United States are of stage III‐IV disease. Despite advancements in radiation techniques and chemotherapy, the overall clinical outcomes for this patient population continue to demonstrate limited improvement,^2^, ^3^ with approximately 15% experiencing locoregional or distant relapse within a five‐year timeframe.^4^, ^5^ Platinum‐based chemotherapy is typically classified as the conventional treatment modality for patients grappling with recurrent or metastatic nasopharyngeal carcinoma (RM‐NPC). Nevertheless, the clinical prognoses for these individuals continue to be unfavorable.^6^ Given the rapid advancements in immunotherapy, Programmed cell death 1 (PD‐1)/Programmed cell death ligand 1 (PD‐L1) inhibitors have emerged as the established first‐line therapeutic option for RM‐NPC.^7^, ^8^, ^9^

Toripalimab, a humanized anti‐PD‐1 antibody, has demonstrated effective anticancer properties in clinical trials. The recent JUPITER‐02 trial, an international, double‐blind, phase 3 trial, revealed that toripalimab plus chemotherapy significantly improved progression‐free survival (median, 11.7 vs. 8.0 months, hazard ratio = 0.52) and overall survival (median OS not reached, hazard ratio = 0.603) in RM‐NPC.^10^ Hence, the United States Food and Drug Administration (FDA) has approved toripalimab in combination with gemcitabine and cisplatin as first‐line treatment for RM‐NPC in October 2023, designating it as an orphan drug for this condition.^11^ This also marks the first approval of a PD‐1 therapy for the treatment of RM‐NPC in the U.S.

Although existing research has provided substantial evidence confirming the safety and efficacy of toripalimab in treating RM‐NPC patients, it is crucial to recognize that the implementation of immunotherapeutic interventions may impose substantial economic burdens on healthcare payers.^12^ Therefore, conducting a comprehensive health economics evaluation is imperative to ascertain its impact on healthcare system payers. There remains a paucity of data regarding the cost‐effectiveness of toripalimab within the American RM‐NPC patient population. The objective of this study is to examine the cost‐effectiveness of toripalimab plus chemotherapy in comparison to chemotherapy regimens for RM‐NPC from the perspective of American payers.

## 2 MATERIALS AND METHODS

### 2.1 Analytical overview

**FIGURE 1:**
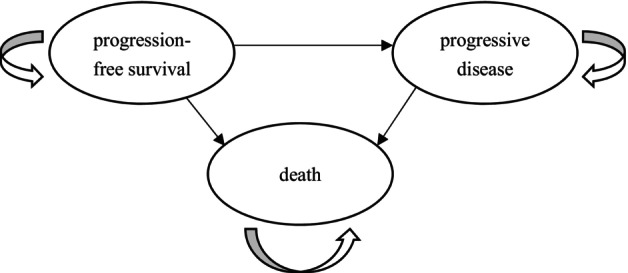
Partitioned survival model structure.

Following the Consolidated Health Economic Evaluation Reporting Standards (CHEERS) 2022 reporting guideline,^13^ a partitioned survival model (PSM) with three health states was developed using Microsoft Excel 2021 to assess the cost‐effectiveness of toripalimab or placebo plus chemotherapy as first‐line treatment in RM‐NPC. As shown in Figure 1, the three mutually exclusive health states: progression‐free survival (PFS), where patients are alive without disease advancement or symptom exacerbation, symbolizing the treatment's success in curbing disease progression; progressive disease (PD), denoting cancer progression and necessitating second‐line treatment due to diminished treatment efficacy; and death, the terminal health outcome reflecting the end of survival analysis and serving to evaluate the mortality risk and the treatment's capacity to extend life. In our model, patients begin in the PFS state and may either transition to PD, move from any state to death, or remain in their initial health state. The PSM determines state membership through two survival curves: the overall survival (OS) curve, which outlines the time from model entry to death, independently establishes the alive and deceased patient proportions over time, disregarding other clinical outcomes and whether patients are in the PFS or PD states. Conversely, the PFS curve tracks the duration from model entry to departure from the PFS state due to disease progression or death, thus defining the PFS state membership over time. State membership for PD is inferred as the residual between the OS and PFS curves, representing the segment of patients who are alive but not in PFS.^14^ This structure allows for a detailed analysis of the interactions between survival, disease progression, and the economic viability of treatments in RM‐NPC. The model time cycle was set at 3 weeks in accordance with the drug regimen. A 30‐year model time horizon was chosen considering the life expectancy of the American population and the average age of diagnosis.^15^ The primary output results of the model consist of quality‐adjusted life year (QALY) and the incremental cost‐effectiveness ratio (ICER). The ICER in our study is calculated using following formula:

$$\text{ICER}=\left({\text{Cost}}_{t}-{\text{Cost}}_{c}\right)/\left({\text{Outcome}}_{t}-{\text{Outcome}}_{c}\right)$$

with ${\text{Outcome}}_{t}$ and ${\text{Outcome}}_{c}$ denoting the cumulative QALYs output in the experimental and control groups, and ${\text{Cost}}_{t}$ and ${\text{Cost}}_{c}$ representing the total costs in the experimental and control groups. A willingness‐to‐pay (WTP) threshold of 150,000 U.S. dollars ($) was adopted as recommended by Neumann.^16^ Both costs and outcomes were discounted at a rate of 3% according to the guidelines.^17^ The evaluation conducted was grounded in a comprehensive literature review utilizing publicly accessible data and established modeling techniques. Given the nature of this secondary data analysis, it did not necessitate review or exemption by an Institutional Review Board or Ethics Committee, aligning with standard protocols for such health economic evaluations.

### 2.2 Population and Interventions

Patient population and regimen in this study were in accordance with the JUPITER‐02 study. Patients aged 18–75 who confirmed primary RM‐NPC after curative therapy were included in our analyses. The dosage regimen was also in conformity with the JUPITER‐02 study. For six cycles, patients were randomized to receive either toripalimab (240 mg) or a placebo together with gemcitabine (2000 mg per m^2^ body surface area) and cisplatin (80 mg per m^2^ body surface area) every 3 weeks. Furthermore, all patients were assumed to receive toripalimab or placebo at the PFS state for up to 2 years or until PD, whichever occurred first. Subsequent treatments were assumed to include best supportive care and pembrolizumab regimen based on the NCCN Clinical Practice Guidelines in Oncology.^18^ The proportions of subsequent treatment regimens and incidence of adverse events (AE) were estimated using information from the supplementary materials of the JUPITER‐02 trial. A mean body surface area of 1.86 m^2^ was used to calculate drug dosages.^19^

### 2.3 Efficacy

The efficacy parameters in partitioned survival model were obtained from the JUPITER‐02 trial by extracting the Kaplan‐Meier (KM) curves of OS and PFS with the GetData Graph Digitizer 2.25, and then we reconstructed individual patient data using Guyot's methodology.^20^ To extrapolate outcomes over the model's time horizon, parametric functions including Weibull, Exponential, Lognormal, Loglogistic, Gompertz, and Generalized Gamma were utilized to fit the pseudo‐survival data. Akaike Information Criterion (AIC), Bayesian information criterion (BIC) and visual inspection were used to gain the optimum model (See Table S1). The final parametric functions of chemotherapy arm's PFS and OS were Log Logistic and Log Normal, respectively. The Log Normal functions provided the best fit for PFS and OS extrapolation for the toripalimab arm (See Figures S1 and S2). The key clinical inputs are presented in Table 1.

**TABLE 1:** Model inputs.

Parameter	Base case	Lower limit	Upper limit	Distribution	Source
Clinical inputs					
Progression‐free survival					
Toripalimab: Log Normal‐Lambda	2.567	2.310	2.824	Lognormal	Extrapolated from published KM curves from JUIPTER‐02
Toripalimab: Log Normal‐Gamma	0.913	0.822	1.005	Lognormal	
Chemotherapy: Log Logistic‐Lambda	2.118	1.906	2.330	Lognormal	
Chemotherapy: Log Logistic‐Gamma	0.341	0.307	0.375	Lognormal	
Overall survival					
Toripalimab: Log Logistic – Lambda	4.094	3.685	4.504	Lognormal	Extrapolated from published KM curves from JUIPTER‐02
Toripalimab: Log Logistic – Gamma	0.701	0.631	0.772	Lognormal	
Chemotherapy: Log Normal – Lambda	3.438	3.095	3.783	Lognormal	
Chemotherapy: Log Normal – Gamma	0.842	0.758	0.927	Lognormal	
Utility inputs					
Toripalimab: PFS	0.68	0.61	0.75	Beta	21
Toripalimab: PD	0.66	0.59	0.73	Beta	21
Chemotherapy: PFS	0.61	0.55	0.67	Beta	21
Chemotherapy: PD	0.47	0.42	0.52	Beta	21
Discount rate, %					
Cost	3	0	8	Beta	17
QALY	3	0	8	Beta	17
Costs inputs (per cycle/ per event), $					
Costs of medication					
Toripalimab	8892	8803	9781	Gamma	22
Cisplatin	33	29	36	Gamma	22
Gemcitabine	73	66	80	Gamma	22
Costs of progressive disease					
Toripalimab	73,143	65,829	80,457	Gamma	18, 22–24
Chemotherapy	88,914	80,023	97,806	Gamma	18, 22–24
Costs of disease management	637	573	701	Gamma	25
Costs of drug administration	651	586	716	Gamma	25, 26
Costs of terminal care	11,126	10,013	12,239	Gamma	26
Costs of adverse events					
Neutropenia	10,073	9066	11,081	Gamma	25
Leukopenia	10,073	9066	11,081	Gamma	25
Anemia	8072	7264	8879	Gamma	25
Thrombocytopenia	9546	8592	10,501	Gamma	25, 27
Lymphopenia	19,805	17,824	21,785	Gamma	26
Hypokalemia	11,889	10,700	13,078	Gamma	27
Hyponatremia	8186	7368	9005	Gamma	27
Pneumonia	16,681	15,013	18,349	Gamma	27

Abbreviations: PD, progressive disease; PFS, progression‐free survival; QALY, quality‐adjusted life year.

Since JUPITER‐02 trial did not report health‐related quality of life (HRQoL) data, we extracted utility values from the input parameters of a published cost‐effectiveness analysis that examined pembrolizumab treatment for advanced recurrent metastatic head and neck cancer in a United States setting. This analysis provided HRQoL data allocated to the PFS and PD states.^21^ In the toripalimab arm, we assumed utility values of 0.68 and 0.66 for the PFS and PD states, respectively. In the chemotherapy arm, corresponding utility values of 0.61 and 0.47 were assumed for the PFS and PD states, respectively.

### 2.4 Cost

All costs were reported in 2022 United States Dollar and adjusted to 2022 values using the Medical‐Care Inflation dataset within Tom's Inflation Calculator.^22^ The model only considered direct costs, including treatment costs, AE management costs, terminal care costs, disease management costs, pharmaceutical management costs, and subsequent treatment costs. Given that the average sales price of toripalimab in the United States has not been disclosed, in our base‐case analysis, we adopted a wholesale acquisition cost.^23^ Price of cisplatin, gemcitabine, and pembrolizumab per unit were acquired from average sales price reported by Centers for Medicare and Medicaid Services.^24^ AEs of grade 1 or 2 often necessitate little or no therapeutic intervention. Consequently, this study selectively focused on the management costs of grade 3 AEs that exhibited an incidence rate exceeding 5%.^25^ AE management costs and terminal care costs were assumed as one‐time costs, with all AEs presumed to occur within the initial treatment cycle. The prices of AE management costs, terminal care costs, disease management costs, and pharmaceutical management costs per cycle were sourced from published literature.^26^, ^27^, ^28^ We considered subsequent treatments as one‐time expenses and estimated the required cycles and associated costs for systemic therapy based on a randomized controlled trial of pembrolizumab as second‐line treatment for RM‐NPC.^29^ Additionally, we calculated the cycles and costs for best supportive care using a systematic review.^30^ The proportions of these approaches applied in the experimental and control groups were obtained from the supplementary materials of the JUPITER‐02 trial. All cost parameters are listed in Table 1.

### 2.5 Sensitivity analyses

We assessed the model's parameter uncertainty through a series of univariate sensitivity analyses. This involved modifying input variables and evaluating their impact on the incremental net monetary benefit (INMB). We calculated the incremental monetary benefit using the following equations:

$$\text{INMB}=\left({\text{Outcome}}_{t}-{\text{Outcome}}_{c}\right)\times \text{Threshold}-\left({\text{Cost}}_{t}-{\text{Cost}}_{c}\right)$$

with Threshold denoting the 150,000 per QALY gained, ${\text{Outcome}}_{t}$, ${\text{Outcome}}_{c}$, ${\text{Cost}}_{t}$, ${\text{Cost}}_{c}$ were defined above.^31^, ^32^ We examined various parameters, encompassing those associated with costs, discount rates for outcomes and costs, and utilities for the PFS and PD states. For most parameters, a ± 10% variation around their base values was applied, whereas discount rate ranges were set from 0% to 8% following previous research.^27^

To address multi‐parameter joint uncertainty, we conducted probabilistic sensitivity analysis with 5000 Monte Carlo simulations. In each iteration, values were randomly sampled for all parameters simultaneously. We employed a lognormal distribution for efficacy parameters, gamma distribution for cost‐related parameters, and beta distribution for health utilities. Table 1 delineates the default values, ranges, and assumed distributions of the model parameters.

In order to investigate the impact of various pricing strategies for toripalimab, we conducted a series of scenario analyses, calculating the ICER for toripalimab at prices ranging from $1000 to $40,000 per‐cycle. To investigate the impact of varying model simulation durations on cost‐effectiveness outcomes, we conducted another scenario analysis, setting the time horizon at 15 years.

Subgroup analyses were conducted to assess the economic benefits of toripalimab plus chemotherapy across various populations. Employing the same methodology as the base case, we extracted the PFS KM curves for the PD‐L1 positive (defined as PD‐L1 positivity ≥1% in tumor cells or immune cells) and PD‐L1 negative (<1%) populations from the Jupiter‐02 trial and fitted parametric equations. Given the absence of reported OS curves for these subgroups in the Jupiter‐02 trial, we assumed their OS KM curves were consistent with the general population.

## 3 RESULTS

### 3.1 Base‐case analyses

In the base case analysis, toripalimab regimen yielded in 4.390 QALYs at a cost of $361,813 Chemotherapy regimen was associated with 1.685 QALYs and a cost of $161,632. This led to an ICER of $74,004/QALY, which is below the WTP threshold of $150,000/QALY. The basic‐case results are shown in Table 2.

**TABLE 2:** Summary of base‐case analyses.

Base‐case	Chemotherapy	Toripalimab	Incremental
Total costs ($)	161,632	361,813	200,181
PFS‐costs of medication	612	175,401	174,789
Costs of drug management	9363	17,649	8286
Costs of disease management	36,873	72,933	36,060
Costs of progressed disease	83,407	63,231	−20,176
Costs of adverse events	21,356	23,974	2618
Costs of terminal care	10,020	8625	−1395
Total QALYs	1.685	4.390	2.705
PFS‐QALYs	0.506	1.063	0.557
PD‐QALYs	1.179	3.327	2.148

Abbreviations: PD, progressive disease; PFS, progression‐free survival; QALY, quality‐adjusted life year.

### 3.2 Sensitivity analyses

**FIGURE 2:**
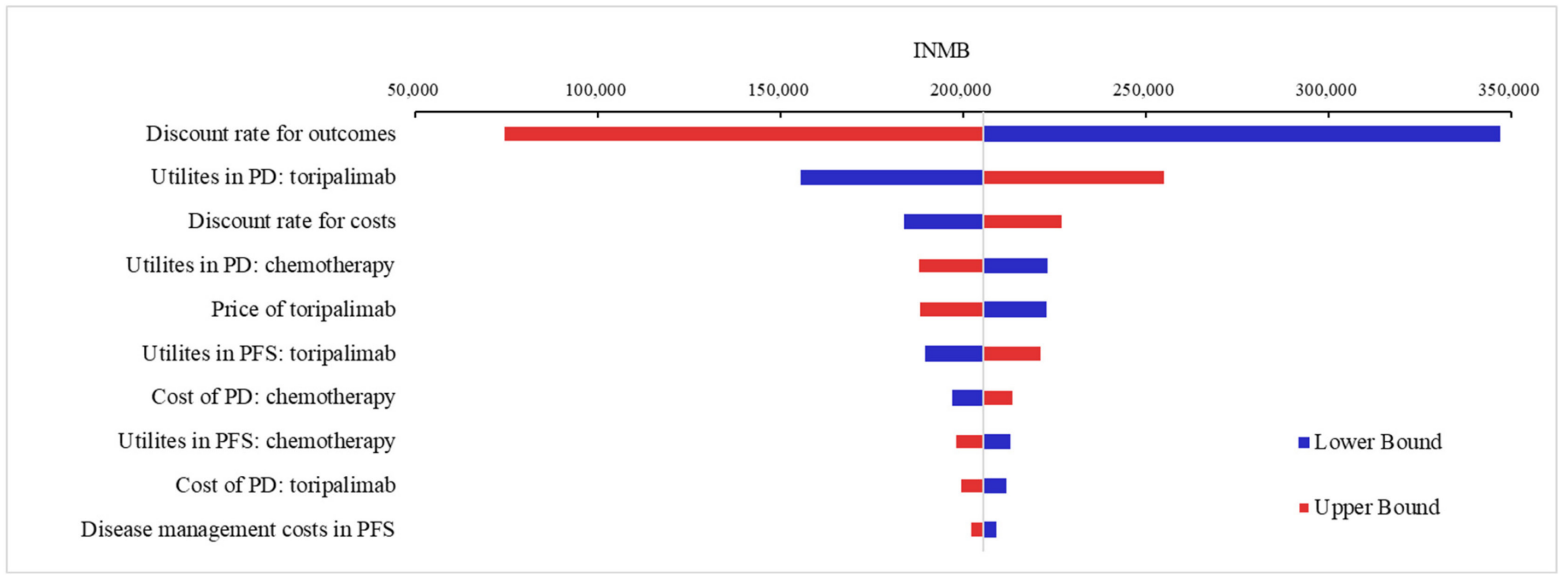
Tornado diagrams of univariable sensitivity analyses. INMB, incremental net monetary benefit; PD, progressive disease; PFS, progression‐free survival.

As shown in Figure 2, univariate sensitivity analyses indicated that parameters of utility values, discount rate, and price of toripalimab have the largest impact on INMB.

The results of the probabilistic sensitivity analysis demonstrated toripalimab plus chemotherapy had an 87.10% probability of being cost‐effective at a threshold of $150,000 compared with chemotherapy alone. If the WTP threshold decreased to approximately $75,000/QALY, there would be a 50% probability that the toripalimab regimen could be considered cost‐effective (See Figure S3 in the Supplement).

**FIGURE 3:**
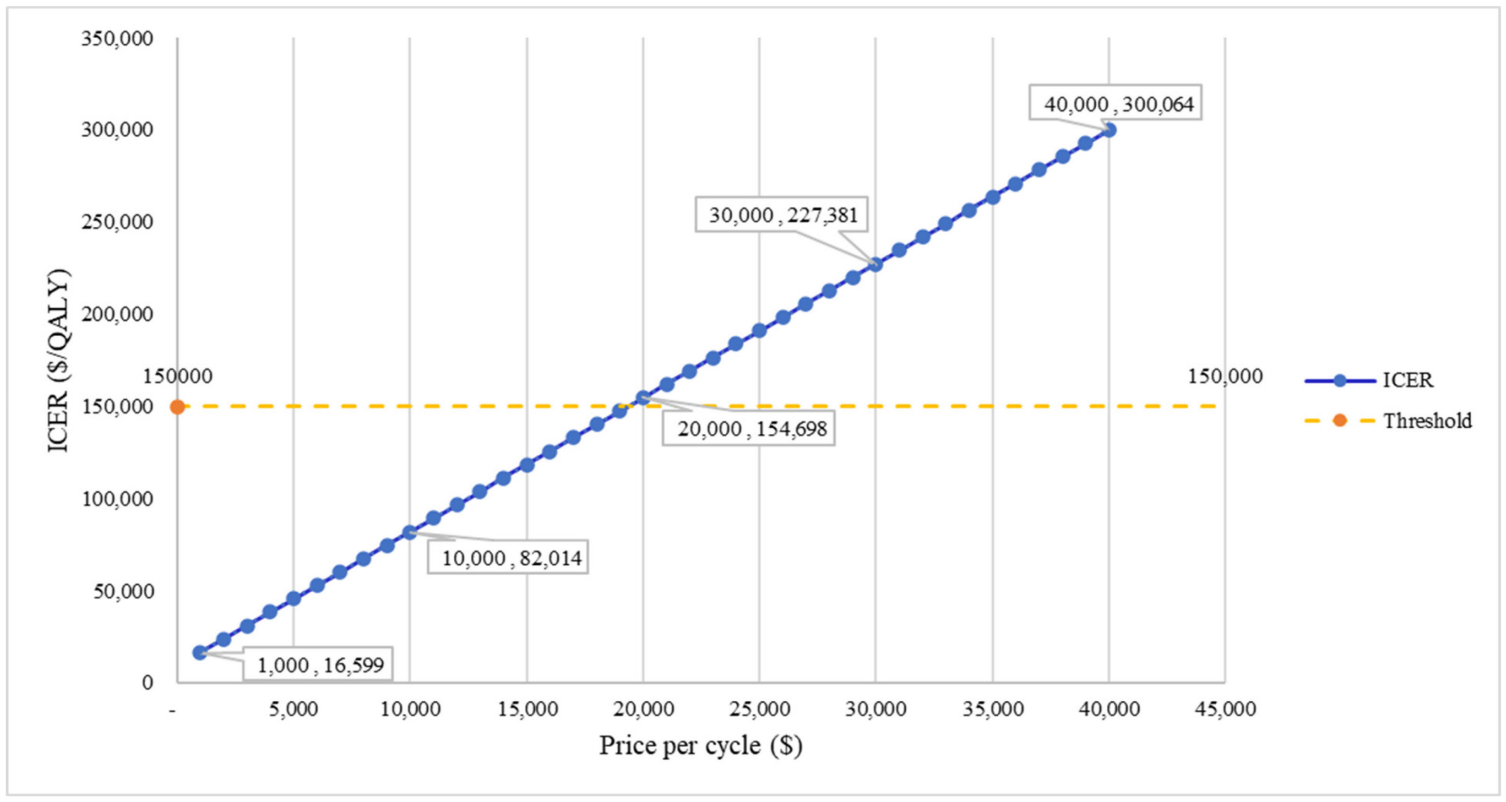
ICER in scenario analysis at various prices. ICER, incremental cost‐effectiveness ratio.

The ICER for each pricing scenario is presented in Figure 3. In the scenario analysis with a price increase of approximately $11,000, the ICER exceeded $150,000, indicating the toripalimab regimen was no longer considered cost‐effective. In the second scenario analysis with a simulated duration of 15 years, the ICER increased from the baseline of $74,004/QALY to $88,026/QALY.

The results of the subgroup analysis revealed ICERs of $76,538/QALY for PD‐L1 positive and $70,158/QALY for PD‐L1 negative groups. (See Table S2). In both nasopharyngeal cancer subgroups, toripalimab treatment remains cost‐effective under a threshold of $150,000 per QALY.

## 4 DISCUSSION

Nasopharyngeal carcinoma patients experience particular obstacles in accessing high‐quality healthcare. In America, patients with NM‐NPC have not benefited from therapeutic advancements for several years, leading to unmet treatment needs. Given the relatively small population of NPC patients in the United States, this group has remained somewhat under the radar in terms of research focus. This underrepresentation highlights a form of health inequality where certain rare conditions may not receive the same level of attention and resources as more prevalent diseases.^33^ Addressing this disparity is essential for promoting health equity and ensuring that all patient populations receive the necessary consideration and support in advancing medical research and care.

Toripaliamb, the first PD‐1 monoclonal antibody approved by the FDA for treating RM‐NPC, meets an unfulfilled clinical demand. The JUIPTER‐02 trial indicated that the toripalimab regimen can offer significant survival benefits for patients, marking a pivotal advancement for RM‐NPC treatment in United States. However, at present, there is insufficient evidence to suggest that toripalimab treatment for nasopharyngeal carcinoma patients is economically acceptable for healthcare payers. Our analysis, the first health economics study for American RM‐NPC patients, suggests that toripalimab regimen exhibit an ICER of $74,004/QALY compared to chemotherapy from the perspective of American payers. Nevertheless, given the substantial survival benefits provided by the toripalimab regimen, to enhance the drug's affordability and make it accessible to a broader patient population, efforts may be made to further reduce the costs.

Univariate sensitivity analyses revealed that the parameters of utility values, discount rate, and price of toripalimab had the most significant impact on INMB. None of these variables could reduce the INMB to less than zero, indicating the robustness of our base case study findings. This result also suggests the long‐term survival benefits of the toripalimab regimen, which are influenced by the discount rate. Furthermore, the lifetime time horizon of our model also amplifies the impact of the discount rate. Due to the utility values for NPC patients in the United States were not available, we were compelled to derive utility values from existing research on American head and neck cancer patients. Given that NPC constitutes a subtype of head and neck cancer, the extrapolation of these utility values is expected to have minimal impact on our outcomes. Furthermore, international studies on the HRQoL of head and neck cancer patients validate this approach, demonstrating a close alignment between the average utility values of the general HNC cohort and the NPC subgroup (0.70 vs. 0.73), affirming the methodological soundness of our utility value estimation.^34^ To assess the impact of toripalimab pricing on ICER, we conducted a series of scenario analyses, encompassing a range of per‐cycle prices from $1000 to $40,000. This extensive analysis substantially mitigated the influence of toripalimab price on the applicability of the study's results. The subgroup analysis indicates that toripalimab is cost‐effective in both PD‐L1 positive and negative patient populations, with greater cost‐effectiveness observed in the PD‐L1 negative group. This finding aligns with the stratified hazard ratio for disease progression reported in the Jupiter‐02 trial (PD‐L1 positive: 0.59 vs. PD‐L1 negative: 0.35).^10^

Previous health economic studies have examined the cost‐effectiveness of toripalimab from the perspective of the Chinese healthcare system,^35^, ^36^, ^37^ which reported ICERs for toripalimab plus chemotherapy versus chemotherapy alone in treating RM‐NPC at $25,576,^35^ $19,726,^37^ and $6696,^36^ demonstrating cost‐effectiveness in China's setting. The divergence between these studies' results and our ICER findings largely originate from the substantial discrepancy in drug pricing between China and the U.S., with the current retail price of toripalimab in America being 34 times that of China's price. Additionally, differences in utility values and other healthcare service costs (e.g., disease management costs) between the two countries also contribute to the variation in ICER values. Besides, these Chinese studies all employed Markov model, as opposed to partitioned survival model, to simulate patients' disease progression. Conversely, our study applied PSM, which does not require the estimation of transition probabilities or the imposition of assumptions on whether death is possible from all health states. According to a recent review, the partitioned survival model is the most commonly used model in NICE oncology drug evaluation reports, accounting for 54% of nearly 100 studies.^38^ Another review based on Canadian Agency for Drugs and Technologies in Health (CADTH) reports and papers published also recommended the partitioned‐survival model is more appropriate when individual data, including reconstructed individual data, are available and the model structure is not complicated.^39^ It is worth noting that, after adjusting utility values and extending the duration of our study simulation, our efficacy outcomes closely resemble those from the three studies mentioned above, which also validates the robustness of our model.

Our study presents several significant advantages. Firstly, to the best of our knowledge, this investigation stands as the initial health economics study within the United States to evaluate treatment strategies for NPC^40^ and serves as a valuable reference for future reimbursement endeavors. Furthermore, our results serve to rectify the lack of attention and dedicated analysis for this specific group of patients, who have long remained in the periphery of clinical and health economic studies. Secondly, our study takes a comprehensive approach by conducting a thorough assessment of the cost‐effectiveness of toripalimab across a spectrum of price points. This analysis equips healthcare insurance providers with essential guidance to inform reasonable payment for toripalimab in America.

Our study bears certain limitations. Firstly, due to the absence of economic burden research on RM‐NPC patients within the United States, we were unable to access data regarding the direct non‐medical and indirect costs incurred by this specific patient group in their pursuit of treatment. As a result, our study was unable to adopt a more comprehensive societal perspective. Secondly, due to the unavailability of original data from the JUIPTER‐02 trial, we had to reconstruct individual patient data from the KM curves of available published articles, which introduce a degree of uncertainty into our study. It is crucial to emphasize that this methodology has gained recognition within the field of health economics.^41^ Furthermore, our study conducted subgroup analyses for PD‐L1 positive and negative populations to explore the economic benefits of toripalimab across various groups. However, due to the limited data reported in literature, we were unable to extend our subgroup analyses to more diverse populations. Should additional data become available, conducting further subgroup analyses in future research will be imperative to enhance the understanding of the intervention's effects across diverse demographic groups. Moreover, it's important to acknowledge that the survival data utilized in our analysis were derived from a randomized clinical trial, which may restrict the applicability of our findings to real‐world settings. Subsequently, given the dearth of economic studies focusing on RM‐NPC within the United States, many of the required cost and utility parameters had to be extrapolated from comparable head and neck cancer populations. To address this limitation, we conducted a series of sensitivity analyses, thus minimizing the potential impact of this approach. Besides, considering variations in cost parameters and the study perspective, the generalizability of our study's cost parameters outside the America is constrained by diverse healthcare economic environments globally. Cost variations, influenced by medical service pricing, labor costs, drug pricing, and national health system strategies, can significantly alter cost‐effectiveness assessments. In countries with different healthcare structures, like single‐payer systems, costs for medical procedures and medications may diverge substantially from American standards due to distinct market regulations and negotiation dynamics. Therefore, the direct application of our American‐based cost findings may not reflect the economic realities or the healthcare cost structure in other countries. While our results offer insights for developed nations with similar healthcare systems, adapting these cost parameters to reflect the specific economic and healthcare contexts of other countries is crucial for accurate cost‐effectiveness analysis.^42^

## 5 CONCLUSIONS

Treatment with toripalimab regimens was likely to be a cost‐effective alternative to standard chemotherapy for American RM‐NPC patients. These findings provide valuable evidence to inform accurate clinical and reimbursement decision‐making for RM‐NPC patients.

## AUTHOR CONTRIBUTIONS

**Dai Lian:** Conceptualization (equal); formal analysis (lead); methodology (lead); writing – original draft (lead). **Yuling Gan:** Software (equal); writing – review and editing (equal). **Dunming Xiao:** Software (equal); writing – review and editing (equal). **Dennis Xuan:** Software (equal); writing – review and editing (equal). **Yingyao Chen:** Conceptualization (equal); writing – review and editing (equal). **Yi Yang:** Conceptualization (equal); writing – review and editing (equal); project administration (lead); resources (lead); supervision (lead).

## FUNDING INFORMATION

This research received no specific grant from any funding agency in the public, commercial, or not‐for‐profit sectors.

## CONFLICT OF INTEREST STATEMENT

The authors made no disclosures.

## Supporting information


Appendix S1.

## Data Availability

Partial data are available in article and readers can contact YY (corresponding author) for other data.
