# Supplementary material for: Toripalimab plus chemotherapy in American patients with recurrent or metastatic nasopharyngeal carcinoma: A cost‐effectiveness analysis
Source: Cancer Med. 2024 May 16;13(10):e7243. doi: 10.1002/cam4.7243 (PMC11097128; doi:10.1002/cam4.7243)
Supplement: Supplementary file 1 — Appendix S1. [file CAM4-13-e7243-s001.docx]

**Table S1.** AIC and BIC Statistics for Alternate Parametric Distributions

| **PFS** | **Toripalimab + chemotherapy ^a^** | | **Chemotherapy** | |
| --- | --- | --- | --- | --- |
| **Fitted Function** | **AIC** | **BIC** | **AIC** | **BIC** |
| Exponential | 247.7623 | 250.7459 | 287.5336 | 290.4964 |
| Weibull | 230.4986 | 236.4659 | 236.6348 | 242.5605 |
| Gompertz | 234.8621 | 240.8293 | 230.1512 | 260.7546 |
| Log logistic | 230.6138 | 236.581 | 230.1512 | 236.0769 |
| Log normal | 232.696 | 238.6632 | 236.3774 | 242.3031 |
| Gen gamma | 232.3516 | 241.3024 | 235.0977 | 243.9863 |
| **OS** | **Toripalimab + chemotherapy** | | **Chemotherapy** | |
| **Fitted Function** | **AIC** | **BIC** | **AIC** | **BIC** |
| Exponential | 171.9267 | 174.9103 | 209.3411 | 212.3039 |
| Weibull | 171.7131 | 177.6803 | 192.8594 | 198.7851 |
| Gompertz | 173.1236 | 179.0908 | 196.9834 | 202.9091 |
| Log logistic | 171.3418 | 177.309 | 192.5592 | 198.4849 |
| Log normal | 171.6240 | 177.5912 | 191.6692 | 197.5949 |
| Gen gamma | 173.3410 | 182.2918 | 193.6649 | 202.5535 |

Abbreviations: AIC, Akaike Information Criterion, BIC, Bayesian information criterion; PD, progressive disease; PFS, progression-free survival. ^a^ In the first cycle, the best fit modeled parametric (Log logistic) of PFS probability is higher than the best fit of OS probability, so the suboptimal distribution (Log normal) of PFS was chosen.

**Table S2.** Summary of Subgroup Analyses

| **Subgroup** | **PD-L1 positive**  **Toripalimab** | **PD-L1 positive**  **Chemotherapy** | **PD-L1 negative**  **Toripalimab** | **PD-L1 negative**  **Chemotherapy** |
| --- | --- | --- | --- | --- |
| Total costs ($) | 368,675 | 162,415 | 351,166 | 159,327 |
| PFS-costs of medication | 181,552 | 610 | 165,973 | 610 |
| Costs of drug management | 18,455 | 10,251 | 16,298 | 6756 |
| Costs of disease management | 72,933 | 36,873 | 72,933 | 36,873 |
| Costs of progressed disease | 63,136 | 83,304 | 63,362 | 83,711 |
| Costs of adverse events | 23,974 | 21,356 | 23,974 | 21,356 |
| Costs of terminal care | 8625 | 10,020 | 8625 | 10,020 |
| Total QALYs | 4.391 | 1.696 | 4.387 | 1.653 |
| PFS-QALYs | 1.112 | 0.554 | 0.982 | 0.365 |
| PD-QALYs | 3.280 | 1.142 | 3.406 | 1.288 |

Abbreviations: PD, progressive disease; PFS, progression-free survival; QALY, quality-adjusted life year.


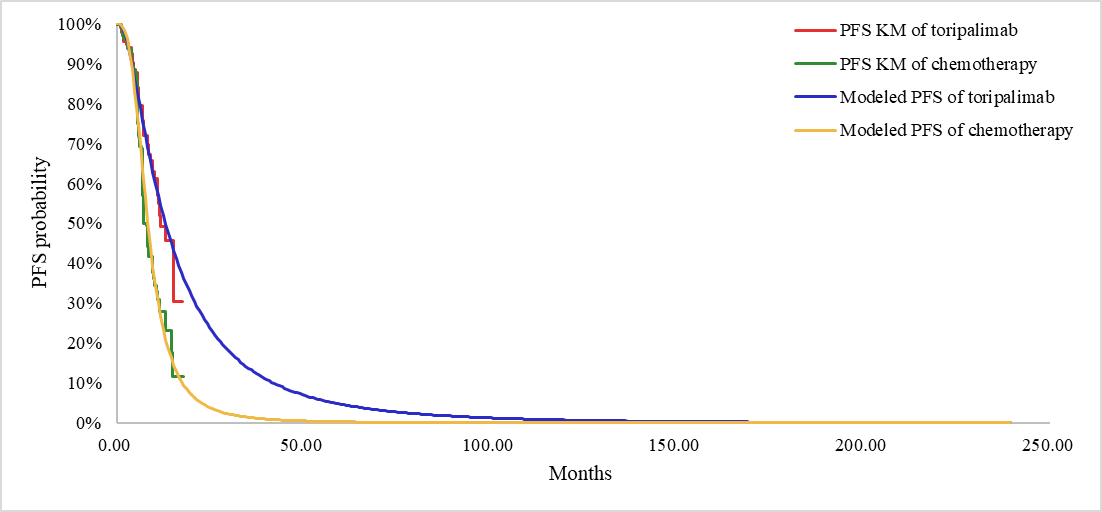
**Figure S1.** Modeled Progression-Free Survival for Toripalimab and Chemotherapy Arms.

**
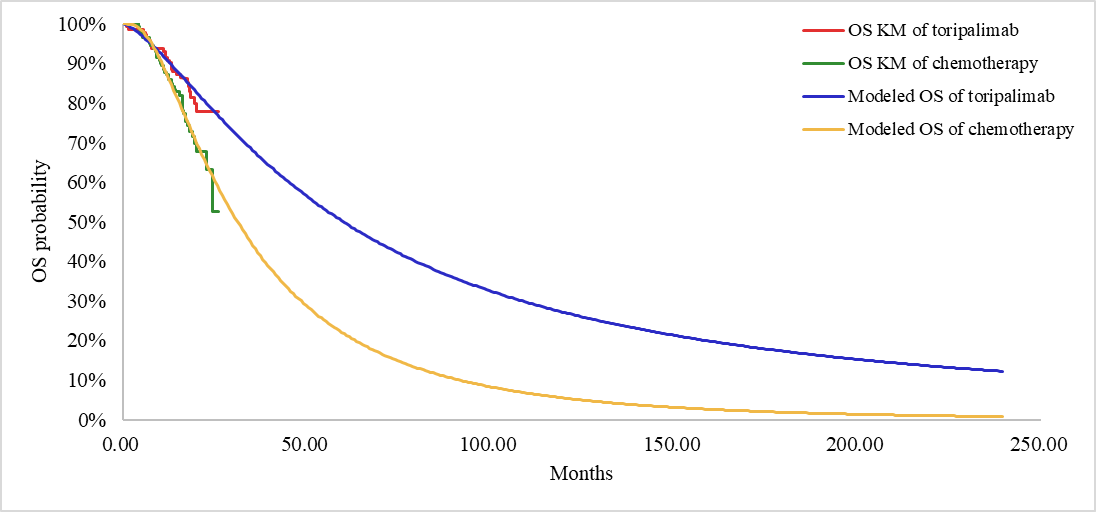
**

**Figure S2.** Modeled Overall Survival for Toripalimab and Chemotherapy Arms.


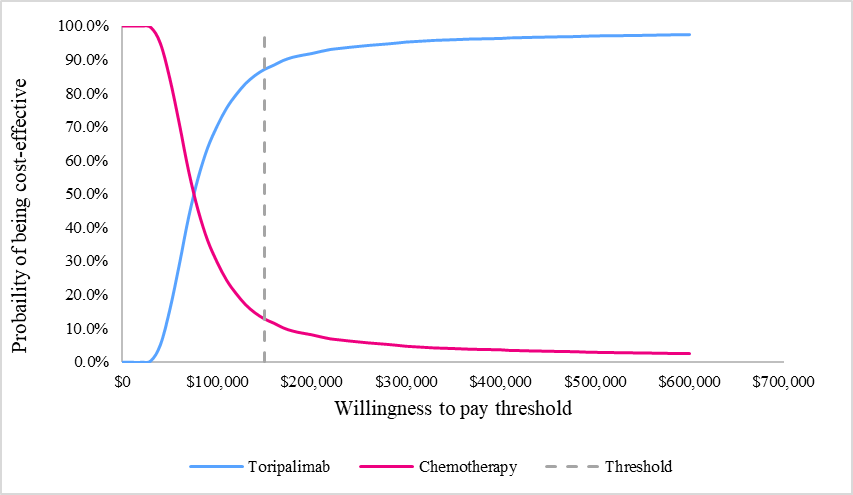


**Figure S3.** Cost-effectiveness Acceptability Curves
